# Supplementary material for: Tryptophanyl-tRNA Synthetase 1 Signals Activate TREM-1 via TLR2 and TLR4
Source: Biomolecules. 2020 Sep 6;10(9):1283. doi: 10.3390/biom10091283 (PMC7565148; doi:10.3390/biom10091283)
Supplement: Supplementary file 1 [file biomolecules-10-01283-s001.zip › Supplementary data.docx]

**SUPPLEMENTARY MATERIALS**

**Figure S1.** (a-c) mRNA levels of (a) IL-1β, (b) TIMP-1, and (c) CXCL2 (IL-8 homolog) in J774.1 cells treated with increasing doses of WARS1 or 500 ng/ml LPS for 8 h.

**Figure S2.** (a) mRNA levels of TREM-1 in THP-1 cells (left panel) or RAW246.7 cells (right panel) treated with increasing doses of WARS1 or 500 ng/ml LPS for 8 h, respectively. (b) Cell lysate (Lys) and cell supernatant (Sup) levels of TREM-1 in THP-1 cells (left panel) or RAW246.7 cells (right panel) treated with increasing doses of WARS1 or 500 ng/ml LPS for 8 h, respectively, were checked by immunoblotting.

**Figure S3.** Coomassie staining (left panel) and endotoxin levels (right table) of the indicated purified proteins used in this study (endotoxin free standard: 0.05 EU/ml).

**Figure S4.** J774.1 cells were treated with PBS, increasing doses of WARS1, or 500 ng/ml LPS for 1, 6, and 12 h, followed by the determination of protein levels using indicated antibodies, respectively.

**Figure S5.** J774.1 cells were treated simultaneously with increasing doses of the Syk inhibitor (R406) and PBS, or 100 nM of WARS1, or 500 ng/ml LPS, for 8 h, and cell viability was measured by WST assay.

**Table S1**. List of primers used in this study.

| **qRT-PCR Primer** | **Primer sequence (5′ to 3′) (Forward)** | **Primer sequence (5′ to 3′) (Reverse)** |
| --- | --- | --- |
| hTREM-1 | GTCTCCACTCCTGACTCTGAA | TAGGGTACAAATGACCTCAGC |
| mTREM-1 | TCCTATTACAAGGCTGACAGAGCGTC | AAGACCAGGAGAGGAAACAACCGC |
| mTREM-2 | GCACCTCCAGGAATCAAGAG | GGGTCCAGTGAGGATCTGAA |
| mTLR4 | GTTGCAGAAAATGCCAGGATG | CAGGGATTCAAGCTTCCTGGT |
| mTLR2 | GGGACGTTTGCTATGATGCC | AAAGGGCGGGTCAGAGTTCT |
| mIL-1β | AGTTGACGGACCCCAAAAGAT | GTTGATGTGCTGCTGCGAGA |
| mCXCL2 | AACATCCAGAGCTTGAGTGTGA | TTCAGGGTCAAGGCAAACTT |
| mTIMP-1 | CCAGAGCCGTCACTTTGCTT | AGGAAAAGTAGACAGTGTTCAGGCTT |
| mMyD88 | CACTCGCAGTTTGTTGGATG | TCTGGAAGTCACATTCCTTGC |
| mDAP12 | TGGTGCCTTCTGTTCCTTCC | TTGTTTCCGGGTCCCTTCC |
| hGAPDH | CACATGGCCTCCAAGGAGTAA | TGAGGGTCTCTCTCTTCCTCTTGT |
| mGAPDH | CGACTTCAACAGCAACTCCCACTCTTCC | TGGGTGGTCCAGGGTTTCTTACTCCTT |
